# The Prophylactic Role of Tranexamic Acid in Reducing Blood Loss During Cesarean Delivery: A Prospective Randomized Controlled Trial

**DOI:** 10.7759/cureus.111875

**Published:** 2026-07-01

**Authors:** Pragya Shree, Roshani Gupta, Divyata Sachan, Vandana Verma, Farha Farahim, Muazzam Hasan

**Affiliations:** 1 Gynecology, Dr. Ram Manohar Lohia Institute of Medical Sciences, Lucknow, IND; 2 Obstetrics and Gynecology, Amar Shaheed Jodha Singh Ataiya Thakur Dariyao Singh Medical College (Autonomous State Medical College, ASMC), Fatehpur, IND; 3 Community Medicine, Hind Institute of Medical Sciences, Barabanki, IND; 4 Obstetrics and Gynecology, All India Institute of Medical Sciences, Raebareli, IND; 5 Maternity and Community Health Nursing, College of Nursing Khamis Mushait, King Khalid University, Abha, SAU; 6 Anesthesia and Critical Care, Amar Shaheed Jodha Singh Ataiya Thakur Dariyao Singh Medical College (Autonomous State Medical College, ASMC), Fatehpur, IND

**Keywords:** elective c-section, emergency cesarean delivery, postpartum hemorrhage, randomized clinical trial, tranexamic

## Abstract

Background: Postpartum hemorrhage (PPH) is a major risk associated with cesarean delivery. This randomized controlled trial (RCT) aimed to evaluate the prophylactic role of intravenous tranexamic acid (TXA) in reducing total intraoperative blood loss in women undergoing lower-segment cesarean section (LSCS).

Materials and methods: The study was conducted at the Autonomous State Medical College, Fatehpur, from September 1, 2024, to March 31, 2025. The primary aim of the trial was to investigate the effect of TXA on perioperative and postoperative blood loss during cesarean section in a low-resource setting. A simple randomization technique was used to allocate 300 participants to either group in a 1:1 ratio.

Results: The median estimated blood loss (EBL) was identical in both groups at 450 mL (IQR, 250-1000 mL), as measured by the gravimetric method. The mean EBL was lower in the TXA group (460.33 mL) than in the placebo group (511.93 mL) (p < 0.05). The mean changes in hemoglobin level and packed cell volume (PCV) were similar between the two groups. There was no statistically significant difference between the groups in the need for additional uterotonics (misoprostol, carboprost, or carbetocin). Blood transfusions were required in 4.7% (7 cases) of the TXA group and 9.3% (14 cases) of the placebo group. Gastrointestinal side effects were reported in 42.0% of the TXA group and 37.3% of the placebo group. No thromboembolic events were reported in either group. The mean birth weight and mean APGAR scores at one minute were comparable between the two groups.

Conclusion: This study supports the prophylactic use of TXA during cesarean delivery to reduce intraoperative blood loss. The significant reduction in mean EBL, combined with the absence of major adverse effects, underscores the role of TXA as a safe and effective adjunct to standard uterotonic therapy.

## Introduction

Cesarean delivery is one of the most common surgical procedures performed worldwide, and postpartum hemorrhage (PPH) remains a leading cause of maternal morbidity and mortality. PPH is a major risk associated with cesarean delivery, accounting for approximately 27% of maternal deaths globally [1]. Excessive bleeding during and after cesarean delivery can lead to serious complications, including hypovolemic shock, organ failure, the need for blood transfusions, and potentially maternal death. Implementing effective strategies to reduce bleeding during cesarean section is essential for improving maternal health, decreasing the need for blood transfusions, and preventing related complications.

The primary pharmacological strategy for preventing and managing postpartum hemorrhage involves the use of uterotonic drugs, with oxytocin being the most commonly administered agent. However, inadequate uterine contractions and activation of the fibrinolytic system can result in excessive bleeding. Tranexamic acid (TXA) has emerged as a promising intervention to reduce bleeding and improve outcomes in cesarean deliveries. TXA, a synthetic derivative of the amino acid lysine, is an antifibrinolytic agent that acts as a competitive inhibitor of plasminogen activation. By preventing the enzymatic degradation of fibrinogen and fibrin clots, TXA stabilizes fibrin clots and reduces hemorrhage [2].

Due to its mechanism of action and proven benefits, TXA has been shown to be highly effective in reducing blood loss and mortality in trauma and general surgery. It is included on the World Health Organization Essential Medicines List for the treatment of postpartum hemorrhage [3]. The administration of TXA during lower-segment cesarean section (LSCS) has emerged as a promising adjunct to standard uterotonic therapy for reducing blood loss [4]. Although numerous studies, including meta-analyses of randomized controlled trials (RCTs), have evaluated this approach, further targeted research is needed to determine the precise extent of the benefits and safety associated with the prophylactic use of TXA [5,6]. Additionally, TXA may be useful in preventing postpartum hemorrhage; however, more evidence is required to fully assess the balance between its risks and benefits.

This RCT aimed to evaluate the prophylactic role of intravenous TXA in reducing total intraoperative blood loss in women undergoing lower-segment cesarean section (LSCS). It also sought to document any adverse events following its administration to provide robust evidence for informing clinical practice guidelines.

## Materials and methods

Study design

This prospective, single-center, parallel-group, placebo-controlled trial was conducted with blinding of participants, operating surgeons, anesthesiologists, and outcome assessors.

Study setting

The study was conducted at the Autonomous State Medical College, Fatehpur, from September 1, 2024, to March 31, 2025.

Ethics and consent

Ethical approval was obtained from the Institutional Ethics Committee (Registration No.: 812/2024-25). The trial was conducted in accordance with the Declaration of Helsinki. The trial was registered with the Clinical Trials Registry of India under registration No. NCT06604325. All patients scheduled for emergency or elective cesarean section were screened for eligibility, and eligible patients were informed about the trial. Written informed consent was obtained from each participant willing to participate in the trial. The study procedures, potential risks, benefits, and data management plan were explained to the participants before they signed the consent forms.

Objective and hypothesis

The primary aim of the trial was to investigate the effect of TXA on perioperative and postoperative blood loss during cesarean section in a low-resource setting.

Research question

A dose of 1 g of intravenous TXA administered 10 minutes before skin incision for elective or emergency cesarean section, compared with placebo, reduces the incidence of postpartum hemorrhage, defined as an estimated blood loss (EBL) of >150 mL within 24 hours of birth.

Hypothesis

H0: The blood loss after cesarean section is similar in both groups.

HA: The blood loss after cesarean section is higher in the control group than in the study group.

Study population

The study was conducted among pregnant women aged 18-45 years who underwent elective or emergency cesarean section.

Inclusion criteria

All pregnant women with singleton pregnancies and gestational ages between 37 and 42 weeks who underwent elective or emergency cesarean section and were willing to participate in the study were eligible for inclusion.

Exclusion criteria

Women who were critically ill or unwilling to participate in the study were excluded. In addition, women diagnosed with uterine rupture, intrauterine fetal death, a history of bleeding disorders, a history of thromboembolism, significant antepartum hemorrhage, or allergy to TXA, as well as those undergoing cesarean section with hysterectomy, were excluded from the study.

Sample size calculation

Based on an 80% study power, an estimated attrition rate of 10%, and a mean difference in blood loss of 197 mL reported in a previous study, the sample size was calculated to be 300 participants (150 in each arm) [5,6].

Study procedure

Randomization and Allocation Concealment

**Figure 1:**
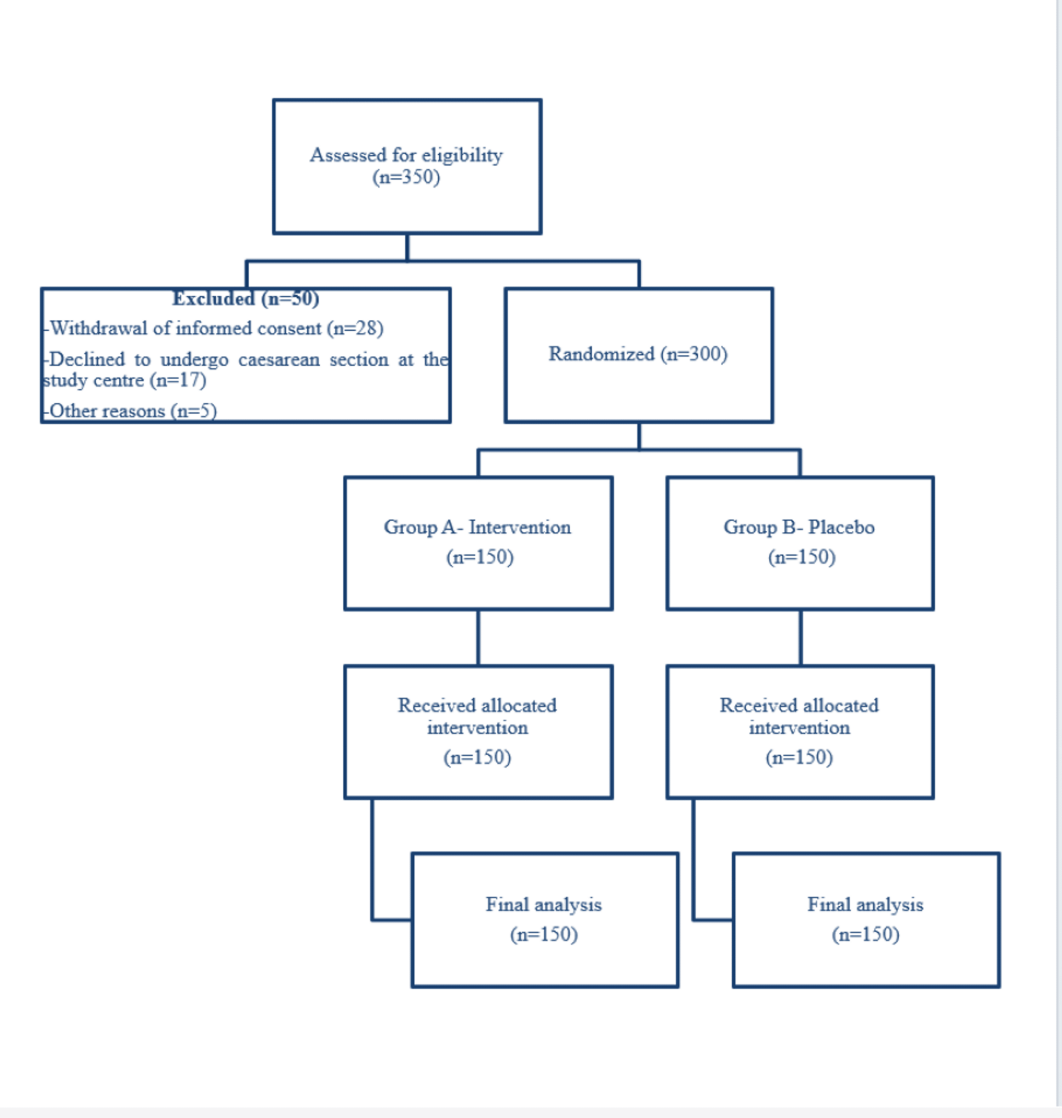
CONSORT flow diagram: participant randomization and treatment CONSORT: Consolidated Standards of Reporting Trials.

Participants who fulfilled the eligibility criteria and provided written informed consent were enrolled in the study. A simple randomization technique was used to allocate participants in a 1:1 ratio to either the TXA group (Group A) or the placebo group (Group B) (Figure 1). Before the initiation of the study, a randomization schedule comprising 300 randomly generated numbers was prepared independently. Each participant was assigned the next sequential random number from this schedule at the time of enrollment. Participants assigned an odd random number were allocated to Group A (TXA), whereas those assigned an even random number were allocated to Group B (placebo).

To ensure allocation concealment, each randomization assignment was placed in a sequentially numbered, opaque, sealed envelope (SNOSE). Immediately before surgery, the next envelope in the sequence was opened only by an independent hospital pharmacist who was not involved in participant recruitment, perioperative management, outcome assessment, or data analysis. According to the treatment allocation indicated inside the envelope, the pharmacist prepared either 1 g of TXA diluted in 100 mL of normal saline or 100 mL of normal saline as a placebo.

To maintain blinding, both preparations were placed in identical infusion bottles and covered with opaque white sleeves before being handed to the anesthesiologist. The participants, operating surgeons, anesthesiologists, investigators, nursing staff involved in intraoperative care, and outcome assessors remained blinded to the treatment allocation throughout the study.

Arms and Intervention

Active comparator (TXA) (Group A): All women recruited to Group A received 1 g of TXA diluted in 100 mL of normal saline as an intravenous infusion administered 10 minutes before skin incision.

Placebo comparator (Group B): All women recruited to Group B received 100 mL of normal saline as an intravenous infusion administered 10 minutes before skin incision.

The assigned intervention for each group is provided in Table 1.

**Table 1:** Arms and interventions

Arms	Assigned interventions
Active comparator (Group A)	Drug: TXA injection. All women recruited to Group A will receive 1 g of TXA in 100 mL of normal saline as an intravenous infusion 10 minutes before the skin incision.
Placebo comparator (Group B)	Drug: Placebo. All women recruited to Group B will receive 100 mL of normal saline as an intravenous infusion 10 minutes before the skin incision.

Surgical Standardization

All cesarean deliveries were performed according to the institution's standardized operative protocol. The surgeries were performed by consultant obstetricians or senior residents under the direct supervision of consultants. Standard lower-segment transverse uterine incisions were used in all patients. Placental delivery, uterine repair, and abdominal closure were performed according to institutional guidelines. Oxytocin was routinely administered after delivery of the baby in both groups, in accordance with hospital protocol. Additional uterotonic agents were administered only when clinically indicated. Postoperative monitoring and management were standardized for all participants.

Blood Loss Measurement

Blood loss was quantified using the gravimetric method from placental delivery until completion of surgery [7]. Before surgery, all surgical mops and drapes were weighed using a calibrated electronic weighing scale. Following surgery, their wet weights were recorded, and the amount of blood absorbed was calculated by subtracting the dry weight from the wet weight (1 g ≈ 1 mL of blood). Blood collected in the suction container after placental delivery was measured separately. Care was taken to minimize contamination from amniotic fluid and irrigation fluids by commencing measurements only after placental delivery and excluding irrigation fluids whenever possible. Total EBL was calculated as the sum of the blood collected in the suction container and the blood absorbed by surgical mops and drapes.

Data collection

Data were collected using a predesigned, pretested, semi-structured questionnaire. The questionnaire was divided into seven sections: sociodemographic profile, obstetric history, details of the current pregnancy, examination findings, intraoperative details, investigations, and postoperative complications.

Outcome measures

The primary aim of the trial was to investigate the effect of TXA on perioperative and postoperative blood loss during cesarean section. Changes in hemoglobin and hematocrit levels were assessed 48 hours postoperatively. Secondary outcome measures included the requirement for additional uterotonics, blood transfusion, or surgical interventions performed intraoperatively for PPH. The effect of TXA on postoperative complications was also evaluated. The Common Terminology Criteria for Adverse Events (CTCAE) was used to classify adverse events (AEs) [8].

Statistical analysis

Statistical analysis was performed using IBM SPSS Statistics for Windows, Version 24.0 (Released 2016; IBM Corp., Armonk, NY, USA). Continuous variables were tested for normality before analysis. Normally distributed variables were expressed as mean ± standard deviation and compared using Student's t-test, whereas categorical variables were expressed as frequencies and percentages and compared using the chi-square test or Fisher's exact test, as appropriate. A two-sided p-value of <0.05 was considered statistically significant.

## Results

As shown in Table 2, the mean age was similar between the groups (TXA: 26.33 ± 3.41 years; placebo: 26.18 ± 3.94 years), with no statistically significant difference (p = 0.956). Similarly, no significant differences were observed between the groups in terms of height, weight, and BMI, with p-values of 0.741, 0.433, and 0.393, respectively. There were slightly more primigravidas in the TXA group (n = 89; 59.3%) than in the placebo group (n = 61; 50.7%). Regarding gestational age, a higher proportion of women in the TXA group were between 37 and 39 weeks of gestation (n = 84; 56.0%) compared with the placebo group (n = 67; 44.7%), whereas a higher proportion of women in the placebo group were beyond 40 weeks of gestation (n = 54; 36.0%) compared with the TXA group (n = 37; 24.7%). However, this difference was not statistically significant (p = 0.078).

**Table 2:** Demographic and clinical profile of participants *Independent t-test. **Chi-square test (df = 197). ***Chi-square test (df = 196).

Characteristics	Subcategory	TXA group (n = 150)	Placebo group (n = 150)	Total (N = 300)	p-value
Age	Mean ± SD	26.33 ± 3.41	26.18 ± 3.94	26.22 ± 3.80	0.956*
	Median (IQR)	26.00 (18-40)	26.00 (19-40)	26.00 (18-40)	
Height (cm)	Mean ± SD	158.93 ± 5.59	158.27 ± 6.19	158.51 ± 5.85	0.741*
	Median (IQR)	160 (142-170)	158 (140-170)	158 (140-170)	
Weight (kg)	Mean ± SD	56.84 ± 7.78	55.30 ± 7.22	55.67 ± 7.66	0.433*
	Median (IQR)	56 (42-82)	54 (42-78)	54 (42-82)	
BMI (kg/m^2^)	Mean ± SD	22.42 ± 2.93	22.04 ± 2.84	22.16 ± 2.87	0.393*
	Median (IQR)	22.0 (16.0-32.0)	21.9 (16.0-32.0)	21.9 (16.0-32.0)	
Parity	Primigravida	89 (59.3)	76 (50.7)	165 (55.0)	0.542**
	Multigravida	61 (40.7)	74 (49.3)	135 (45.0)	
Gestational age (weeks)	34-36	29 (19.9)	29 (19.3)	58 (19.3)	3.662***
	37-39	84 (56.0)	67 (44.7)	151 (50.3)	
	>40	37 (24.7)	54 (36.0)	91 (30.3)	

Table 3 shows the distribution of maternal complications among the study participants. Anemia was the most common complication, affecting 31.33% (n = 47) of participants in the TXA group and 36.00% (n = 54) in the placebo group. Hypertensive disorders of pregnancy were the second most common complication, occurring in 21.33% (n = 32) of the TXA group and 17.33% (n = 26) of the placebo group. Other complications included gestational diabetes mellitus, eclampsia, and intrahepatic cholestasis. Overall, the rates of maternal complications were comparable between the two groups.

**Table 3:** Distribution of maternal complications among study subjects

Distribution of maternal complication	TXA group (n = 150)	Placebo group (n = 150)	Total (n = 199)	Chi-square value		p-value
Anemia	47 (31.33)	54 (36.00)	101 (67.33)	0.7314 (df = 1)	0 .617	
Hypertensive disorders of pregnancy	32 (21.33)	26 (17.33)	58 (38.67)	0.7694 (df = 1)	<0.05	
Gestational diabetes mellitus	17 (11.33)	19 (12.66)	36 (17.12)	0.1264 (df = 1)	0.246	
Eclampsia	4 (2.67)	9 (6.00)	13 (8.67)	2.0102 (df = 1)	0.257	
Intrahepatic cholestasis	8 (5.33)	11 (7.33)	19 (12.67)	0.5056 (df = 1)	0.246	

The indications for cesarean delivery were compared between the TXA and placebo groups (Table 4). Fetal indications were significantly more common in the placebo group (n = 112; 74.7%) than in the TXA group (n = 88; 58.7%) (p < 0.05). Non-progress of labor also differed significantly between the groups, occurring in 36.0% (n = 54) of the TXA group and 50.7% (n = 76) of the placebo group (p < 0.05). Other indications, including maternal medical complications, previous cesarean section, oligohydramnios/polyhydramnios, and abnormal presentation, were comparable between the two groups. Overall, the study suggests that the TXA group had lower rates of fetal complications and non-progress of labor compared to the placebo group.

**Table 4:** Distribution of indications for cesarean among study participants

Indication for cesarean delivery	TXA group ( n = 150)	Placebo group (n = 150)	Chi-square value	p-value
Maternal medical disorders	6 (4.0)	7 (4.7)	0.0804 (df = 1)	0.246
Oligohydramnios/polyhydramnios	5 (3.3)	3 (2.0)	0.5136 (df = 1)	0.777
Previous cesarean section	26 (17.3)	30 (20.0)	0.3514 (df = 1)	0.720
Fetal complication	88 (58.7)	112 (74.7)	8.64 (df = 1)	0.553
Abnormal presentation	30 (20.0)	27 (18.0)	0.1948 (df = 1)	0.768
Non-progress of labor	54 (36.0)	76 (50.7)	6.57 (df = 1)	0.000079

The median EBL was identical in both groups at 450 mL (interquartile range (IQR): 250-1000 mL) (Table 5). However, the mean EBL was lower in the TXA group (460.33 mL) than in the placebo group (511.93 mL), and this difference was statistically significant (p < 0.05). Although the medians and IQRs were identical, a statistically significant difference was observed in the mean EBL. The mean change in hemoglobin level was similar between the two groups (0.99 ± 1.61 g/dL in the TXA group vs. 0.97 ± 1.12 g/dL in the placebo group; p = 0.097) (Table 5). The mean change in packed cell volume (PCV) was also comparable between the groups (3.43 ± 4.94% in the TXA group vs. 3.49 ± 4.56% in the placebo group; p = 0.913). These differences were not statistically significant. The median change in hemoglobin was slightly lower in the TXA group (0.75 g/dL) than in the placebo group (0.90 g/dL). Similarly, the mean change in PCV was slightly lower in the TXA group than in the placebo group. There was no statistically significant difference between the groups in the requirement for additional uterotonics (misoprostol, carboprost, or carbetocin). However, a statistically significant difference was observed in the need for additional surgical management: six patients (4.0%) in the TXA group required surgical intervention, whereas none in the placebo group required surgery (p < 0.05). Blood transfusions were required in 4.7% (n = 7) of the TXA group and 9.3% (n = 14) of the placebo group. Although the proportion of patients requiring blood transfusion was lower in the TXA group, the difference between the groups was not statistically significant (p = 0.175).

**Table 5:** Distribution of study participants according to blood loss and the need for additional intervention

Characteristics	Subcategory	TXA group (n = 150)	Placebo group (n = 150)	Chi-square test value	p-value
Estimated blood loss	Mean ± SD	460.33 ± 129.71	511.93 ± 164.13	1.950 (df = 197)	<0.05
	Median (IQR)	450 (250-1000)	450 (250-1000)		
Change in Hb (g/dL)	Mean ± SD	0.97 ± 1.12	0.99 ± 1.61	0.243 (df = 196)	0.907
	Median (IQR)	0.75 (1.10-4.20)	0.90 (3.40-5.00)		
Change in PCV (%)	Mean ± SD	3.43 ± 4.94	3.49 ± 4.56	0.354 (df = 196)	0.913
	Median (IQR)	3.1 (9.7-18.9)	2.6 (5.6-18.9)		
Additional uterotonic	Misoprost	5 (3.3)	12 (8.0)	1.702 (df = 1)	0.134
	Carboprost	12 (8)	14 (9.3)	0.026 (df = 1)	0.837
	Carbetocin	6 (4.0)	7 (4.7)	0.012 (df = 1)	1.000
Additional surgical management		6 (4.0)	0 (0.0)	2.866 (df = 1)	<0.05
Blood transfusion until hospital discharge		7 (4.7)	14 (9.3)	0.712 (df = 1)	0.175

The study evaluated the adverse effects in the TXA and placebo groups (Table 6). The results showed that 58.0% (n = 87) of participants in the TXA group and 62.67% (n = 94) in the placebo group did not experience any adverse effects. Gastrointestinal side effects were reported in 42.0% (n = 63) of participants in the TXA group and 37.33% (n = 56) in the placebo group. No thromboembolic events were reported in either group.

**Table 6:** Distribution of adverse effects noted among study participants

Adverse effects	TXA group (n = 150)	Placebo group (n = 150)	Chi-square test value	p-value
None	87 (58.0)	94 (62.67)	0.6826 (df = 4)	0.765
Gastrointestinal side effects	63 (42.0)	56 (37.33)		
Thromboembolic events	0 (0.0)	0 (0.0)		

As shown in Table 7, the neonatal outcomes demonstrated that the mean birth weight was slightly lower in the TXA group (2.68 ± 0.54 kg) than in the placebo group (2.97 ± 0.23 kg). The mean Apgar score at one minute was comparable between the two groups, with the TXA group having a mean score of 8.32 ± 0.26 and the placebo group having a mean score of 8.41 ± 0.28. At five minutes, the mean Apgar scores were also similar, with the TXA group having a mean score of 9.23 ± 0.35 and the placebo group having a mean score of 9.45 ± 0.47. Overall, neonatal outcomes were comparable between the two groups.

**Table 7:** Distribution of neonatal outcomes among study participants

Neonatal outcome	TXA group	Placebo group	Chi-square test value	p-value
Mean birth weight in kg (±SD)	2.68 ± 0.54	2.97 ± 0.23	6.0513 (df = 298)	0.654
Mean APGAR score at one minute	8.32 ± 0.26	8.41 ± 0.28	2.8848 (df = 298)	0.702
Mean APGAR score at five minutes	9.23 ± 0.35	9.45 ± 0.47	4.5980 (df = 298)	0.348

## Discussion

In 2017, the World Maternal Antifibrinolytic (WOMAN) trial demonstrated that early administration of the antifibrinolytic drug TXA, within three hours after childbirth, reduces deaths due to PPH and decreases the need for surgical interventions to control bleeding without increasing the risk of vascular occlusive events. Shortly thereafter, TXA was incorporated into the World Health Organization (WHO) guidelines for the management of PPH [9,10]. Based on the dosing regimen used in the WOMAN trial, the WHO recommends administering 1 g of TXA intravenously, followed by a second 1 g intravenous dose if bleeding persists after 30 minutes or recurs within 24 hours. TXA is affordable, stable at high temperatures, and has a long shelf life. A key advantage of TXA is its ability to reduce PPH-related mortality regardless of the underlying cause of bleeding.

This RCT evaluated the prophylactic role of intravenous TXA in reducing intraoperative blood loss and the incidence of PPH among women undergoing LSCS. The baseline characteristics, including maternal age, height, weight, BMI, and gestational age, were comparable between the TXA and placebo groups, confirming that the study groups were well matched and that the observed outcomes were attributable to the intervention rather than demographic differences.

The primary outcome of this study was EBL. The mean EBL was significantly lower in the TXA group (460.33 mL) than in the placebo group (511.93 mL), despite similar median values (450 mL). This statistically significant reduction in mean EBL suggests that prophylactic administration of TXA effectively reduces intraoperative blood loss during cesarean delivery. These findings are consistent with previous studies by Preeti et al., Hemapriya et al., and Sinha et al., who also reported significant reductions in blood loss with prophylactic TXA use during LSCS [11-13]. The antifibrinolytic mechanism of TXA, which prevents the conversion of plasminogen to plasmin and stabilizes fibrin clots, likely underlies this benefit.

Although the mean reductions in hemoglobin and PCV were slightly smaller in the TXA group than in the placebo group, these differences were not statistically significant. This may reflect the modest absolute reduction in blood loss, which, although statistically significant, was not large enough to result in clinically meaningful changes in hematological parameters. In contrast, Shalaby et al. reported a statistically significant reduction in perioperative hemoglobin levels in the placebo group compared with the intervention group [14]. A recent systematic review and meta-analysis by Bellos and Pergialiotis [15] and Abu-Zaid [16] reported changes in hemoglobin favoring TXA with moderate-quality evidence, whereas Lee et al. reported similar findings specifically in women undergoing cesarean delivery [17].

The need for blood transfusion was lower in the TXA group (4.7%) than in the placebo group (9.3%), although this difference did not reach statistical significance. This trend is clinically relevant, as reduced transfusion requirements may minimize exposure to transfusion-related risks and reduce the burden on healthcare resources. The requirement for additional uterotonic agents was similar between the groups, suggesting that TXA may act synergistically rather than redundantly with uterotonic therapy. A systematic review and meta-analysis of RCTs reported a reduced need for blood transfusion in both low-risk and high-risk patients; however, the quality of evidence was low [18]. An unexpected finding was the higher frequency of additional surgical interventions in the TXA group. Because only six participants required additional procedures and no thromboembolic events or coagulation abnormalities were identified, this observation is likely attributable to chance rather than a direct effect of TXA. Nevertheless, this finding warrants cautious interpretation and should be explored in larger, adequately powered multicenter studies.

Regarding safety, TXA administration was well tolerated. No thromboembolic events were recorded, and the incidence of gastrointestinal side effects (nausea and vomiting) was comparable between the TXA and placebo groups. These findings support the favorable safety profile of TXA in obstetric practice, as corroborated by large meta-analyses and the WOMAN trial (2017), which confirmed the absence of excess thrombotic complications associated with TXA use in the postpartum period [9].

However, the WOMAN-2 trial reported that, in women with moderate-to-severe anemia, administration of TXA within 15 minutes after umbilical cord clamping did not reduce the incidence of clinically diagnosed PPH [19].

The Tranexamic Acid to Reduce Blood Loss in Hemorrhagic Cesarean Delivery (TRACES) trial reported that an intravenous dose of 1 g, rather than 0.5 g, of TXA is optimal for inhibiting the fibrinolytic pathway in the treatment of PPH [20].

The findings of the present study are consistent with recent RCTs demonstrating that prophylactic TXA reduces intraoperative blood loss during cesarean delivery. However, similar to several previous studies, improvements in hematological indices and transfusion requirements were not consistently demonstrated. Therefore, although prophylactic TXA appears effective in reducing measured blood loss, the clinical significance of this reduction should be interpreted cautiously.

Neonatal outcomes were reassuring in both groups. Mean birth weights and Apgar scores at one and five minutes were comparable, indicating that maternal administration of TXA before cesarean delivery does not adversely affect neonatal well-being. These findings are consistent with previous studies and systematic reviews demonstrating that TXA does not cross the placenta in clinically significant amounts or adversely affect neonatal outcomes [13,21,22].

Strengths and limitations

The strengths of this study include its randomized controlled design, blinded administration of the intervention, standardized perioperative management, and comprehensive assessment of maternal and neonatal outcomes. Blood loss was measured using a standardized gravimetric technique, and all participants underwent uniform postoperative follow-up.

Several limitations should be acknowledged. First, this was a single-center study, which may limit the external validity of the findings. Second, although randomization minimized selection bias, minor baseline imbalances in the indications for cesarean delivery were observed and may have independently influenced intraoperative blood loss. Third, gravimetric estimation of blood loss may have been affected by contamination with amniotic fluid or irrigation fluid, despite efforts to minimize measurement error. Fourth, the study was not powered to detect differences in uncommon adverse events or secondary clinical outcomes, such as blood transfusion requirements. Finally, elective and emergency cesarean deliveries were analyzed together without stratified subgroup analysis, which may have influenced the observed treatment effect.

## Conclusions

Prophylactic intravenous TXA administered before cesarean delivery was associated with a statistically significant reduction in mean intraoperative blood loss compared with placebo. However, no statistically significant differences were observed in postoperative hemoglobin decline, hematocrit changes, blood transfusion requirements, or major maternal and neonatal adverse outcomes.

These findings suggest that TXA may be a useful adjunct to standard uterotonic therapy for reducing measured blood loss during cesarean delivery. Nevertheless, the clinical significance of the observed reduction should be interpreted cautiously, as several secondary outcomes did not differ significantly between the groups and the study was conducted at a single center. Larger multicenter RCTs employing robust allocation methods and stratified analyses are warranted to further establish the effectiveness and safety of prophylactic TXA during cesarean delivery.
